# Invasive Pulmonary Aspergillosis Presenting as a Giant Abscess in a Post-tuberculosis Destroyed Lung: A Case Report

**DOI:** 10.7759/cureus.105173

**Published:** 2026-03-13

**Authors:** Joke Goethals, Robina Aerts, Abraham Van Poppel, Talli Naamani

**Affiliations:** 1 Department of Pulmonology, University Hospital of Antwerp, Antwerp, BEL; 2 Faculty of Health Sciences, University of Antwerp, Antwerp, BEL

**Keywords:** anti-aspergillus igg antibody elisa, chronic pulmonary aspergillosis, invasive aspergillosis, post-tuberculosis lung disease (ptld), real time aspergillus pcr

## Abstract

Invasive pulmonary aspergillosis (IPA) is a severe fungal infection caused by *Aspergillus *mold, primarily affecting immunocompromised individuals, but increasingly seen in those with critical illness (e.g., viral infections) or in a subacute form in those with severe lung disease. We report the case of a 38-year-old woman with a history of pulmonary tuberculosis (TB) and a suspicion of post-TB chronic pulmonary aspergillosis who presented with dyspnoea and a productive cough unresponsive to antibiotics. Her condition rapidly evolved to respiratory insufficiency requiring intubation. Imaging revealed an extensive abscess in the left upper lobe with surrounding consolidation. Bronchoscopy showed a large amount of mucus requiring clearance. Cytopathology and cultures confirmed *Aspergillus fumigatus *(*A. fumigatus*). Laboratory testing showed marked eosinophilia, elevated total immunoglobulin-E, and positive *A. fumigatus *precipitins. The combination of a predisposing factor (post-tuberculosis cavities), cultures, and radiological findings was compatible with the diagnosis of subacute invasive pulmonary aspergillosis or chronic cavitating aspergillosis, as strictu sensu diagnosis of probable invasive aspergillosis could not be made (as typical immunocompromising host factors were not met). However, the disease was rapidly progressive, and the patient exhibited a good clinical, biochemical, and radiological evolution under voriconazole therapy. Long-term antifungal therapy will be required. This case highlights the importance of considering IPA, even in patients without classic profound immunosuppression, and recognizing post-tuberculosis cavities as a significant predisposing factor.

## Introduction

Chronic pulmonary aspergillosis (CPA) is a progressive respiratory infection that largely occurs in immunocompetent or subtly immunocompromised individuals with underlying structural lung diseases, most commonly treated tuberculosis (TB) [1,2]. Following treatment for pulmonary TB, a significant proportion of patients, estimated to be between 20% and 40%, have residual lung cavities. These persistent pulmonary spaces create a niche susceptible to fungal colonization, which often results in the formation of a mycetoma, or aspergilloma (a fungal ball within the cavity). This colonization can lead to the expansion of existing cavities and subsequent surrounding lung damage, thus establishing the chronic, destructive nature of CPA [3]. While it is not the most common pathway, an evolution or transition from the chronic, destructive nature of CPA to the acute, invasive nature of invasive pulmonary aspergillosis (IPA) is definitely possible if the patient's immune status significantly declines [4].

Histopathological demonstration of fungal tissue invasion remains the diagnostic gold standard for invasive aspergillosis. However, tissue sampling is frequently not feasible in routine clinical practice. As delayed diagnosis is associated with increased mortality, it is crucial to rapidly achieve an accurate diagnosis by integrating microbiological and histopathological results with host factors and clinical-radiological evidence [5].

We present a case of a rapidly aggressive pulmonary aspergillosis with a history of an aspergilloma within a post-tuberculosis cavity.

## Case presentation

**Figure 1:**
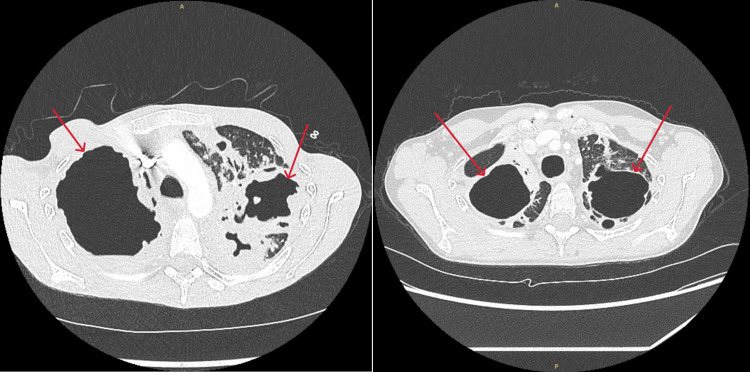
Computed tomography of the chest showing cavities before (left) and after (right) tuberculosis treatment.

**Figure 2:**
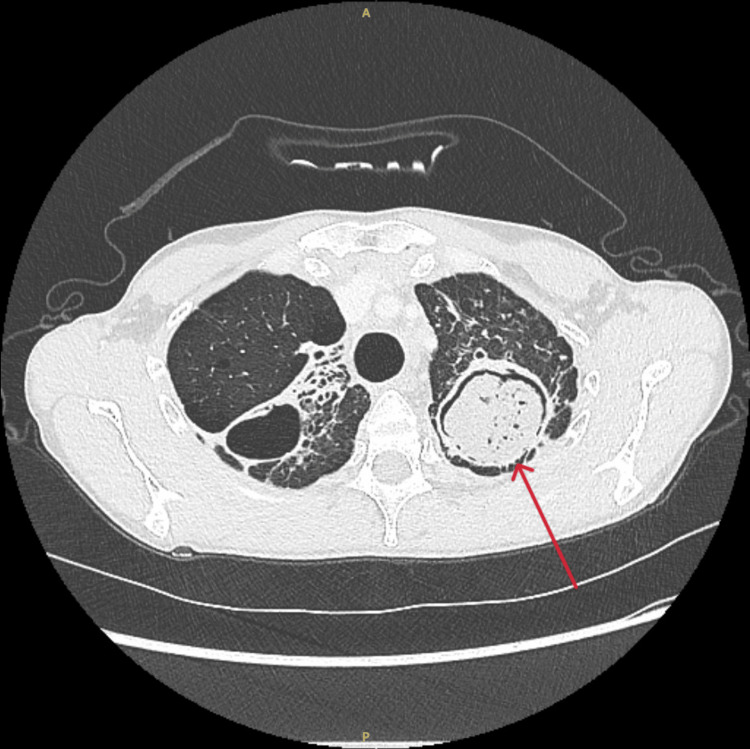
Computed tomography of the chest shows an aspergilloma in the left upper lobe.

A 38-year-old woman, an active smoker (>5 pack years), with a history of pulmonary tuberculosis (see Figure 1) and osteomastoiditis (diagnosed based on radiological findings in combination with positive auramine stain and cultures, treated with quadruple therapy in the form of isoniazid, rifampicin, pyrazinamide and ethambutol in 2021) and a recently suspected chronic pulmonary aspergillosis (based on an aspergilloma, see Figure 2), presented ambulatorily with dyspnoea and a productive cough. She was further known with obstructive lung disease for which she was treated with a combination inhaler containing a long-acting beta-agonist (LABA) and a long-acting muscarinic antagonist (LAMA). A course of amoxicillin-clavulanic acid was initiated based on sputum cultures positive for *Staphylococcus aureus*. As she showed no improvement a few days later, and due to associated fever, night sweats, weight loss, and thoracic pain, she was referred to the emergency department for further evaluation.

**Figure 3:**
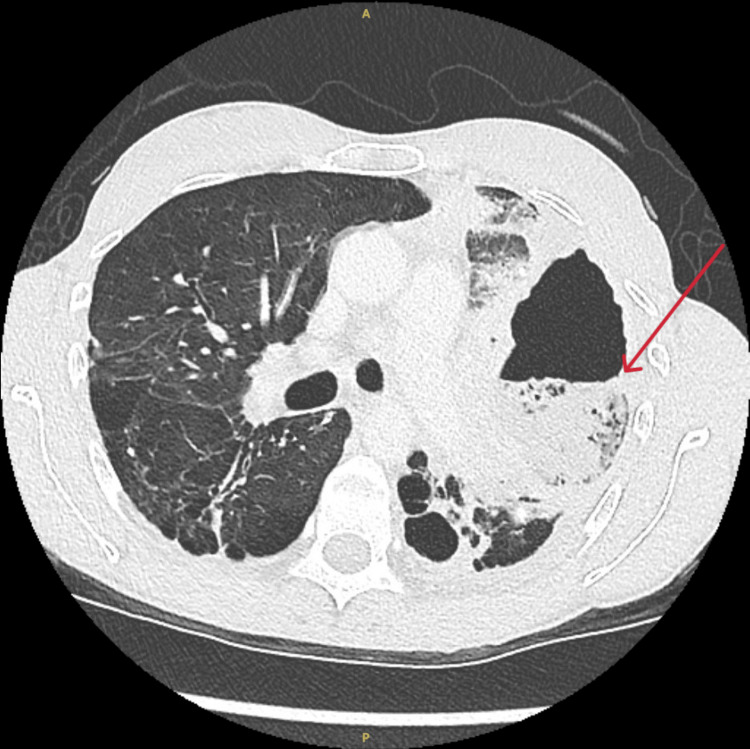
Computed tomography of the chest shows an abscess in the left upper lobe containing an air-fluid level.

On examination, auscultation was normal, but she reported painful palpation of the left chest. Laboratory findings showed severely elevated inflammatory markers (C-reactive protein 393 mg/dL, normal <5) and eosinophilia of 680/µL (absolute count, normal <250). Initial chest computed tomography (CT) showed an extensive pulmonary abscess (9.6 x 7.1 x 10.5 cm) in the left upper lobe containing fluid/pus with an air-fluid level (Figure 3), as well as increased consolidation in the left upper lobe. Increased mediastinal lymph nodes were also noted, deemed likely reactive. Due to the clinical deterioration and radiological findings, therapy was escalated to piperacillin-tazobactam and voriconazole based on a high suspicion of subacute invasive pulmonary aspergillosis (IPA).

Shortly after admission to the pulmonology department, the patient had to be transferred to the intensive care unit due to respiratory insufficiency. Initially, oxygen via high-flow nasal cannula (Optiflow, Fisher & Paykel Healthcare, Auckland, New Zealand) was started, but she required intubation shortly afterward.

A control X-ray showed a decrease in the fluid collection, suggesting spontaneous drainage. Subsequently, a therapeutic bronchoscopy was performed, including the evacuation of a large amount of pus from the bronchi and a bronchoalveolar lavage (BAL). Aerobic cultures initially remained negative, but further results showed a positive galactomannan (Platelia Aspergillus enzyme immunoassay, Bio-Rad, Hercules, California, USA, ODI 7.82, normal <0.5), Aspergillus PCR (AsperGenius, PathoNostics, Maastricht, Netherlands), and eventually positive cultures for *Aspergillus fumigatus*. Molecular testing confirmed the absence of common cyp51A mutations (e.g., TR34/L98H). Additional blood testing showed an Aspergillus-specific Immunoglobulin G (IgG) of 815 mg/L (normal <72) and a total Immunoglobulin E (IgE) of 2135 kE/L (normal <114). Screening for HIV and hepatitis A, B, C, and E was negative. Immunoglobulin levels and flow cytometry results, obtained outside of the acute infectious episode, were also within normal limits. Results for *Mycobacterium tuberculosis* (PCR) were only weakly positive, and all sputum auramine stains were negative. These microbiological and laboratory findings are summarized in Table 1. Microbiology confirmed a very low likelihood of active tuberculosis, pointing instead toward a past infection. Consequently, we opted against starting tuberculostatics or maintaining respiratory isolation. Based on host, culture, and radiological findings, a probable invasive aspergillosis was stated.

**Table 1:** Summary of laboratory findings Decreasing Aspergillus IgG and galactomannan under treatment, suggesting a good outcome. ODI: Optical Density Index

Test	Time of diagnosis	Follow-up
Sputum culture	Enterobacterales , Staphylococcus aureus	Not performed
Sputum culture with fungal culture requested	Aspergillus fumigatus, Streptococcus agalactiae, Auramine staining negative, PCR for Mycobacterium tuberculosis (M. tuberculosis) positive, PCR Rifampicin resistance negative	Not performed
Bronchoalveolar lavage fluid (BAL)	Galactommanan ODI 7.82, Aspergillus fumigatus, Streptococcus agalactiae, Auramine staining negative, PCR for M. tuberculosis negative, Aspergillus PCR not performed	Galactommanan ODI 4.13, Fungal culture negative, Streptococcus agalactiae, PCR for M. tuberculosis weakly positive, Auramine staining negative, Mycobacterial culture negative, Aspergillus PCR positive (genotypic resistance testing negative)
Cytological analysis of BAL fluid	Neutrophilic granulocytic inflammation, Grocott staining positive (some rare septated hyphae)	Not performed
Blood testing	Aspergillus IgG 815 mg/L (normal value <72 mg/L), Total IgE 2135 kE/L (normal <114)	Aspergillus IgG 189 mg/L (normal value <72 mg/L)

**Figure 4:**
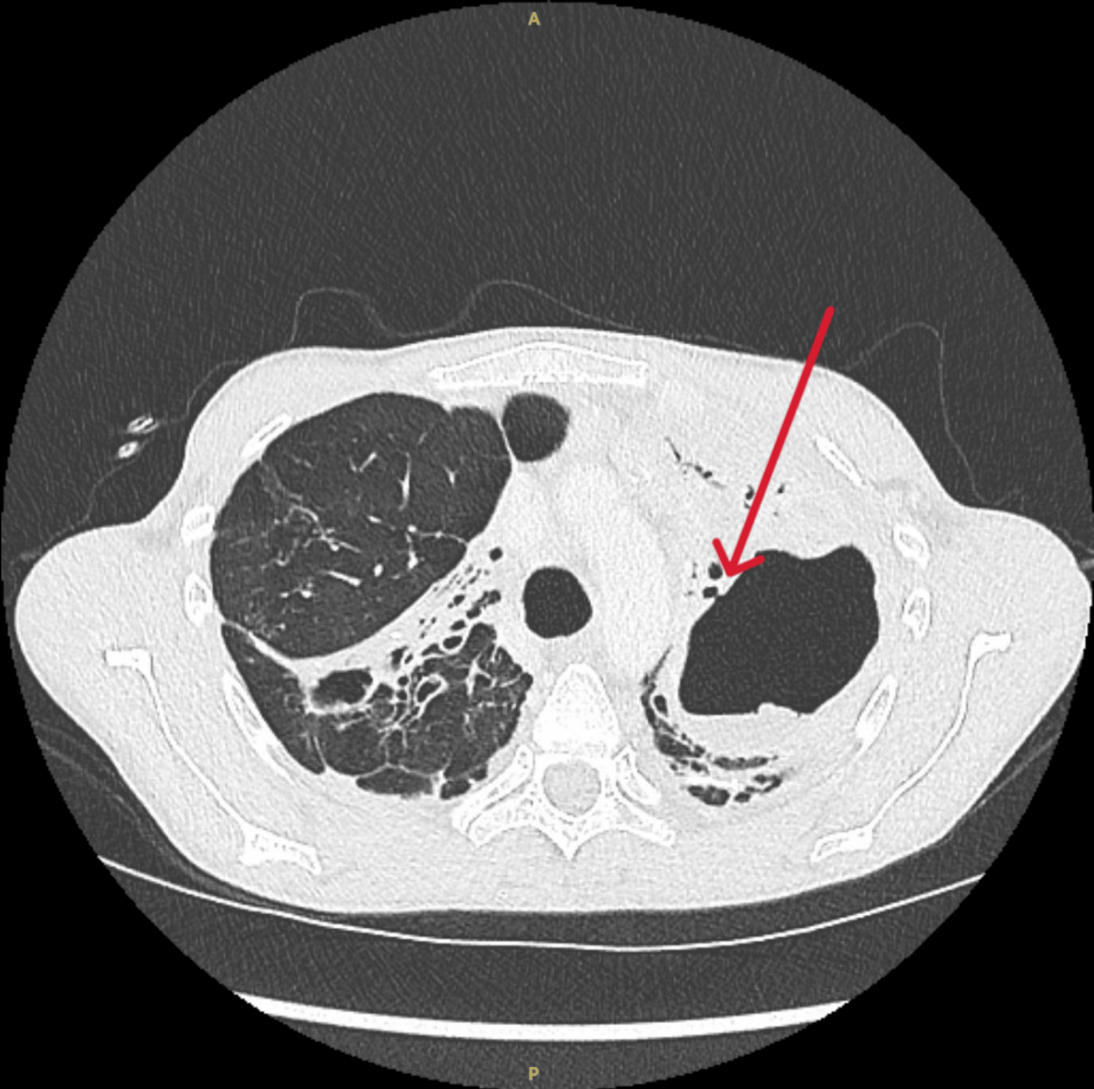
Computed tomography of the chest with one of the three fistulae.

Under the implemented management plan, there was a favorable evolution, after which extubation was possible, and the patient could be transferred back to the pulmonology ward. A control CT confirmed the suspected drainage by visualizing three small fistulous connections from the collection to branches of the upper lobe bronchus (Figure 4). Antibiotic treatment was stopped after two weeks, while voriconazole was continued. The patient was ultimately discharged home after optimizing the dose of voriconazole.

**Figure 5:**
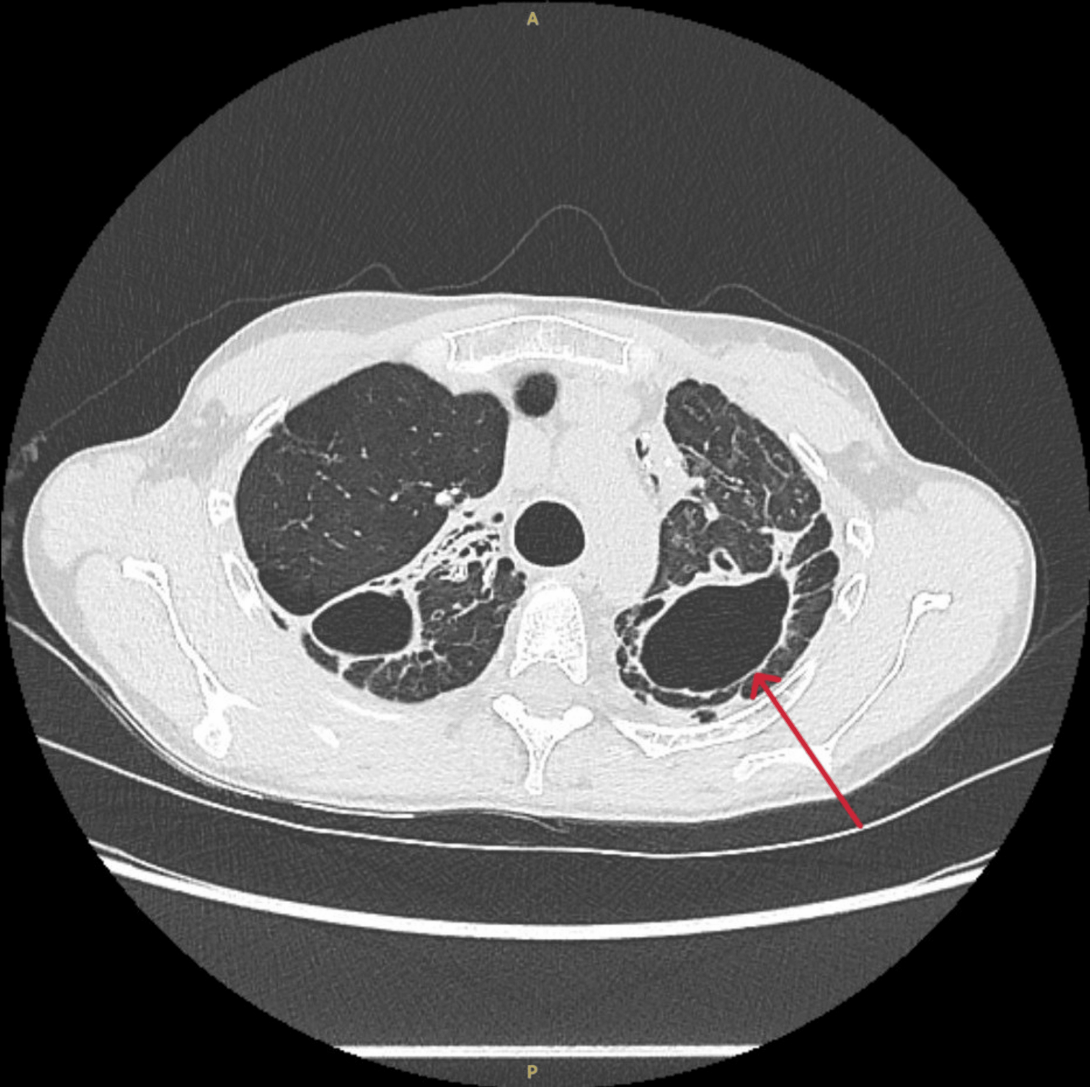
Computed tomography of the chest shows shrinkage of the abscess with a residual cavity.

Four months after discharge, the patient is doing well. CT showed a clear volume reduction of the pulmonary abscess in the left upper lobe, with currently mainly a residual cavity (Figure 5). Additionally, there were decreased consolidations and ground-glass opacities in both lower lobes. Aspergillus IgG was also decreasing (189 mg/L). The follow-up microbiological and laboratory findings are summarized in Table 1. Despite the absence of enzyme-inducing drug interactions, voriconazole concentrations remained highly variable and frequently subtherapeutic (ranging from 0.5 to 2.2 mg/L) even after dose escalation to 400 mg twice daily. Given this instability and the need for long-term therapy, the patient was switched to isavuconazole.

Further follow-up is planned after three months with a CT scan, blood samples, and lung function testing. Treatment with isavuconazole is still being continued, and there is still no known end date of therapy because of the underlying chronic component. A close follow-up has been established.

## Discussion

Diagnosing pulmonary aspergillosis (PA) remains challenging because its key clinical and radiological features often overlap with those of active pulmonary tuberculosis (PTB) or PTB relapse. As previously suggested, a considerable number of PTB relapses may have represented underdiagnosed fungal infections. A persistent cough, weight loss, and lung cavitation are common to both conditions, frequently leading to PA being misdiagnosed and inappropriately treated as "smear-negative PTB." Furthermore, low clinical awareness and limited access to specific diagnostics contribute to this error. Clinicians must therefore maintain a high suspicion and rule out other cavitary infections, including non‑tuberculous mycobacteria (NTM) and endemic mycoses, when managing patients with persistent cavitary lung disease [2,6].

Our case report is a rare and diagnostically challenging presentation of subacute invasive pulmonary aspergillosis occurring in a patient with a unique predisposing factor: an aspergilloma sitting inside a residual post-tuberculosis cavity. It's well-known that following successful PTB treatment, residual lung cavities persist in a substantial number of patients.

These structural defects create an ideal environment for *Aspergillus* colonization, typically resulting in chronic pulmonary aspergillosis, where the fungus forms a mycetoma. While IPA is typically reserved for profoundly immunocompromised hosts, this case illustrates the overlap, suggesting a transition from the chronic, localized CPA to a rapid, destructive, and invasive disease. This presentation, often termed subacute invasive aspergillosis (SAIA) or chronic necrotizing pulmonary aspergillosis (CNPA), is driven by the severe structural damage and is commonly seen in patients with moderate immune impairment, where the pathology behaves more aggressively than classic CPA but slower than acute IPA [4,7]. Customized definitions have been proposed for IPA in ICU, and specifically for IPA in patients with influenza or COVID-19, but when these host factors are missing, the criteria are not met. However, suggested classifications are mostly set up for study purposes, not to guide clinicians [8-10]. This case, reflecting a rapidly progressive disease, is to be treated as an invasive infection.

Given the complexity of this case, sitting between established chronic colonization and acute invasion, a rapid, multi-modal diagnostic approach was necessary. The serological results provided important context: the presence of high Aspergillus IgG and IgE levels strongly suggested a history of long-standing colonization or CPA/allergic bronchopulmonary aspergillosis (ABPA) features. While serum galactomannan was not assessed in this case, it is worth noting that in subacute or chronic forms of pulmonary aspergillosis, serum levels are frequently negative, unlike in acute invasive disease. However, the high galactomannan levels in bronchoalveolar fluid, combined with the patient's acute respiratory failure and the extensive consolidation seen on imaging, confirmed the rapid, invasive, and life-threatening nature of the current episode. Given the severe prognostic implications of delayed antifungal intervention, achieving a rapid, accurate diagnosis is paramount. This goal is met not through reliance on isolated results, but by strategically integrating the clinical presentation, radiological imaging, and supporting microbiological data [7,11]. Also, this patient’s chronic obstructive lung disease was a contributing condition to the development of pulmonary aspergillosis [12,13].

The immediate management involved the aggressive treatment typically reserved for acute IPA, using systemic azoles, in consideration of local antifungal resistance rates. Azole resistance rates in Belgium are increasing and approach 10%. In this case, azole resistance testing was performed on positive *Aspergillus* cultures as well as by Aspergillus PCR [14]. The favorable clinical and radiological evolution under this therapy supports the diagnosis of an invasive process that was effectively treated. However, this case carries significant long-term concerns. Even after the acute infection is controlled, the patient remains at high risk for relapse because the primary host factor, the residual PTB cavity, persists. Therefore, the management strategy must transition from acute IPA treatment to a long-term strategy aimed at controlling the underlying CPA, often requiring prolonged antifungal therapy to prevent disease recurrence, further structural damage, and subsequent invasive episodes. This mandates continuous surveillance and follow-up to optimize the patient's long-term outcome [7,15].

## Conclusions

In conclusion, this case report details a complex presentation of rapidly progressive pulmonary aspergillosis (PA) arising in a patient with an underlying post-tuberculosis lung cavity complicated by a prior aspergilloma. The patient's rapid deterioration and need for intensive care underscored the high mortality risk associated with (sub)acute fungal disease. The clinical picture highlights the potential for chronic colonization, facilitated by structural lung damage, to transition into an acute, invasive infection, even without classic profound immunosuppression. This necessity for rapid diagnosis, achieved through the prompt synthesis of clinical, radiological, and microbiological data (host factors, imaging, and biomarkers), is paramount for initiating treatment on time and securing a favorable prognosis. Ultimately, this report emphasizes the critical importance of maintaining a high index of suspicion for aspergillosis in all patients with chronic cavitary lung disease.
